# Efficient ^15^N hyperpolarization of [^15^N_3_]metronidazole antibiotic via spin-relayed pulsed SABRE-SHEATH

**DOI:** 10.1016/j.jmro.2025.100208

**Published:** 2025-08-02

**Authors:** Shiraz Nantogma, Shannon L. Eriksson, Thomas Theis, Warren S. Warren, Boyd M. Goodson, Eduard Y. Chekmenev

**Affiliations:** a Department of Chemistry, Integrative Biosciences (IBio), Karmanos Cancer Institute (KCI), Wayne State University, Detroit, MI 48202, USA; b Department of Chemistry, School of Medicine, Duke University, Durham, NC 27708, USA; c Department of Chemistry, North Carolina State University, Raleigh, NC 27695-8204, USA; d Department of Chemistry, Biomedical Engineering, and Radiology, School of Medicine, Department of Physics, Duke University, Durham, NC 27708, USA; e School of Chemical & Biomolecular Sciences and Materials Technology Center, Southern Illinois University, Carbondale, IL 62901, USA

**Keywords:** Hyperpolarization, Nitrogen-15, Sabre, Parahydrogen, Metronidazole

## Abstract

Signal Amplification by Reversible Exchange in SHield Enables Alignment Transfer to Heteronuclei (SABRE-SHEATH) is an NMR hyperpolarization technique that relies of the simultaneous exchange of parahydrogen and a to-be-hyperpolarized molecule on the metal center of a polarization-transfer catalyst in a microtesla magnetic field. Until recently, this method has been understood to perform hyperpolarization by establishing level anti-crossings between the nuclear spins of the parahydrogen derived hydrides (acting as a source of hyperpolarization) and those of the substrate. Recently, the application of highly non-intuitive pulse sequences (comprising pulses of microtesla DC fields) was predicted to hyperpolarize nuclear spins more efficiently than the canonical (static-field) SABRE-SHEATH approach. Here we show that by employing a basic “on-off” pulse sequence of rectangular microtesla pulses, it is possible to improve the hyperpolarization efficiency for SABRE-SHEATH of [^15^N_3_]metronidazole, an FDA-approved antibiotic (in non-enriched and non-hyperpolarized form) and potential hypoxia sensing molecule. Specifically, we demonstrate that ^15^N polarization of 18.5 % can be obtained in 80 s of parahydrogen bubbling parahydrogen through a solution containing 20 mM [^15^N_3_]metronidazole. In practice, (1.32 ± 0.14)-fold improvements in $P_{15N}$ was obtained with the pulsed method described here compared to static field technique variant. These results show that pulsed SABRE-SHEATH was successfully applied to ^15^N-labeled biologically relevant molecule. Moreover, we also demonstrate that although the pulsed SABRE-SHEATH sequence was designed for polarization transfer from parahydrogen derived hydrides to the metronidazole’s ^15^N catalyst-binding site, all three ^15^N sites of [^15^N_3_]metronidazole attained the hyperpolarized state. This spin-relayed polarization transfer becomes possible due to the ^15^N relay network established by their spin-spin *J*-couplings. The feasibility of the spin-relayed polarization transfer is demonstrated here for the first time for pulsed SABRE-SHEATH (as opposed to the static-field SABRE-SHEATH reported previously) and it paves the way to broad applicability of the technique.

## 1. Introduction

One of the major driving forces of research in the magnetic resonance community is the search for novel molecular contrast agents that can yield new information about physiological and pathological conditions [1]. Hyperpolarization is one of the most promising methods for increasing NMR signals, and enabling molecular imaging of low concentrations of hyperpolarized (HP) metabolites [2–6]. Spin Exchange Optical Pumping (SEOP) [7] and dissolution Dynamic Nuclear Polarization (d-DNP) [8,9] are two widely used hyperpolarization techniques. Both of them have progressed to clinical trials. HP ^129^Xe gas was approved by the FDA for functional lung imaging in late 2022. However, these hyperpolarization techniques generally employ complex, expensive and low-throughput equipment, and alternative hyperpolarization techniques that can provide higher throughput at lower cost are under development [10–12]. One such alternative method is Signal Amplification by Reversible exchange (SABRE) [13]. SABRE exploits the singlet spin order inherent in parahydrogen (pH_2_), which is a readily available source of nuclear spin order in this technique [14]. A to-be-hyperpolarized molecule and pH_2_ bind transiently to the metal center of a catalyst [14]. During this chemical exchange, a transient polarization transfer (PTC) complex is formed [13], transferring the spin order from pH_2_-derived hydride protons to the target nuclear spins via spin-spin *J*-couplings [13]. Following polarization transfer, the HP substrate and H_2_ molecules are released from the catalyst metal center [15]. The average lifetime of the PTC species is on the order of 10^−2^ s, and when polarization transfer is repeated over many seconds, then this process leads to polarization build-up of the “free” molecules of the substrate in the solution [14]. While there are other parahydrogen-based methods such as hydrogenative Parahydrogen Induced Polarization (PHIP) [16–19], SABRE has the clear advantage that substrate molecules are hyperpolarized via reversible chemical exchange, i.e., without chemical modification of the substrate [20], making the hyperpolarization process easily repeatable.

The original design of the SABRE experiment performed in millitesla static magnetic fields was later rationalized via levels anti-crossings (LACs) that enable spontaneous polarization transfer between the pH_2_-derived hydrides and substrate protons, when a magnetic field is matched with corresponding values of the spin-spin couplings and the frequency differences of the nuclei participating in SABRE polarization transfer [21]. Theis and co-workers have later demonstrated that the LACs to transfer SABRE-based hyperpolarization to heteronuclei (i.e., anything but protons, e.g., ^13^C and ^15^N), are found at microtesla magnetic fields: typically 0.1 – 1 μT [22–24]. This demonstration gave rise to SABRE in SHield Enables Alignment Transfer to Heteronuclei (SABRE-SHEATH) [23,24], an acronym for the SABRE experiment performed in microtesla fields for spontaneous and efficient polarization transfer from pH_2_-derived hydrides to ^13^C [25], ^15^N [22–24], and other heteronuclear nuclei [26]. Since then the work in the field has focused on expanding the range of molecular targets to a wide range of small molecules and biologically relevant molecular targets [27], development of biocompatible formulations [28–31], and improving the efficiency of polarization transfer from the pH_2_-derived singlet to target nuclei of interest [32–36].

Recently, Lindale, Eriksson, and co-workers [34,37] demonstrated a pulsed SABRE-SHEATH process that applies microtesla fields to the SABRE sample in discrete pulses instead of a continuous static applied field in order to make the polarization transfer more coherent. This approach was shown to increase ^15^N polarization by a factor of 1.3 for [^15^N]acetonitrile [38], although simulations have predicted over a factor of 3 increase in the expected $P_{15N}$ in that model spin system [34]. Moreover, first pilot studies employing pulsed SABRE-SHEATH to [1–^13^C]pyruvate yielded no improvement over the conventional static-field SABRE-SHEATH approach [39].

In this work, we explore the utility of the pulsed SABRE-SHEATH for the hyperpolarization of [^15^N_3_]metronidazole (MNZ). Metronidazole is an antibiotic that belongs to the class of nitroimidazoles [40]. HP [^15^N_3_] metronidazole has the potential to be used for molecular imaging of hypoxia due to its structural similarity to Positron Emission Tomography (PET) agents such as ^18^F-fluoroazomycin arabinoside (^18^F-FAZA) and fluoromisonidazole (^18^F-FMISO) [40–43]. The nitro group of this class of compounds is reduced during cell metabolism, in particular in low-oxygen environments, i.e., under hypoxic conditions [44]. Following uptake, downstream metabolites get trapped within cells and the radioactive accumulation [44] is imaged, which can serve as a diagnostic marker for solid tumors using such PET radiotracers [45–47]. Recent computational studies revealed that chemical shifts of HP [^15^N_3_] metronidazole could be employed to report on the stepwise reduction of metronidazole in hypoxic environments, and therefore act as a contrast agent of hypoxia [48]. Moreover, feasibility of detecting of HP [^15^N_3_] metronidazole in the brain of healthy rats has been established recently [48], paving the way for future use of this HP contrast agent exhibited ultra-long ^15^N *T*_1_ of up to 10 mins at 1.4 T [48–50].

Previous SABRE-SHEATH studies have reported $P_{15N}$ in excess of 20 % for natural-abundance (0.4 %) ^15^N in metronidazole [51,52]. Somewhat lower $P_{15N}$ values of 12–15 % were reported for fully ^15^N labeled [^15^N_3_]metronidazole [49,53], which was rationalized through a larger ^15^N payload and a larger spin-bath in this triply labeled metronidazole variant. Here, we investigate the feasibility of pulsed SABRE-SHEATH to hyperpolarize fully labeled [^15^N_3_]metronidazole using a basic rectangular “on-off” pulsing of the DC microtesla field, to investigate the polarization dynamics. Besides focus on a different molecule from recently studied [1–^13^C]pyruvate [39], the work reported here targets ^15^N (vs. ^13^C) nucleus with substantially longer lasting HP state in AA’XX’ spin system (vs. AA’X in case of [1–^13^C]pyruvate [39]) and also investigates the efficiency of spin-relayed polarization transfer under conditions of pulsed fields.

We find that pulsed SABRE-SHEATH approach yields (1.32 ± 0.14)-fold greater $P_{15N}$ on all three labeled ^15^N sites compared to continuous static SABRE-SHEATH with the same compound. Our pilot results also pave the way to overall more efficient ways of SABRE-SHEATH hyperpolarization for other biologically relevant ^15^N molecules including [^15^N_3_]nimorazole, [^15^N]dalfampridine [54], ornidazole [55], and others [56–58].

## 2. Materials and methods

### 2.1. Sample preparation

**Fig. 1.**
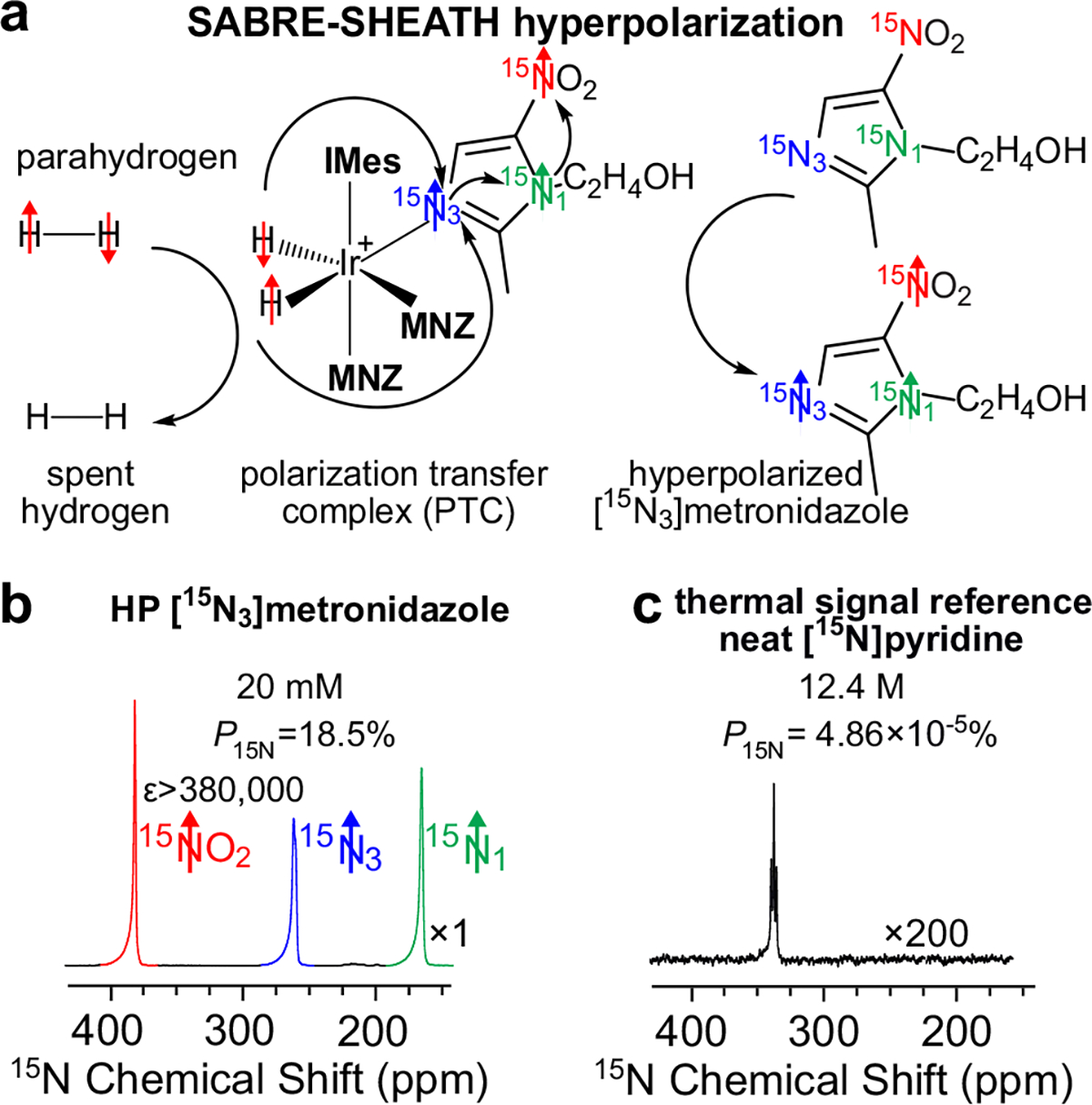
a) Schematic of simultaneous chemical exchange of pH_2_ and [^15^N_3_] metronidazole (MNZ) with the activated IrIMes catalyst complex to yield HP [^15^N_3_]metronidazole based on previous studies [49,53] (note the Cl^−^ counter ion is not shown). b) ^15^N NMR spectrum of 20 mM HP [^15^N_3_]metronidazole prepared via pulsed SABRE-SHEATH (pH_2_ flow of 70 sccm at 8 atm; τ_LOW_=4 ms; τ_HIGH_=2.4 ms; *B*_LOW_<40 nT; *B*_HIGH_=−28 μT) using 2 mM IrCl(COD)(IMes) pre-catalyst in CD_3_OD at room temperature (data shown corresponds to the maximum $P_{15N}$ obtained in the experimental series shown in Figure S1c). c) Corresponding ^15^N spectrum of the signal reference compound (neat [^15^N] pyridine) recorded using 64 scans and repetition time of 10 min.

40 mM [^15^N_3_]metronidazole and 4 mM pre-catalyst (IrCl(COD) (IMes); COD = cyclooctadiene, IMes = 1,3-bis(2,4,6-trimethyl-phenyl) imidazole-2-ylidene)) [15,59] were dissolved in CD_3_OD contained in two separate plastic Eppendorf vials. 0.30 mL of each solution was then taken and mixed together in a 5-mm medium-wall NMR tube to make a resulting 0.60-mL solution, containing 20 mM [^15^N_3_]metronidazole and 2 mM of the SABRE pre-catalyst. The NMR tube was jacketed with ¼”-outer diameter (OD) Teflon tubing. The solution was flushed via bubbling with ultra-high-purity Argon gas for 2 minutes to remove any trapped air. Next, the jacketed medium-wall NMR was connected to a hyperpolarizer setup via a wye connector [60]. The sample was then activated by bubbling with pH_2_ gas for 2 h in the NMR tube at a flow rate of 20 standard cubic centimeters per minute (sccm) and 8 bar pressure to yield the SABRE-active complex shown in Fig. 1a [53]. Please, note that although activation is a slow process, this solvent composition and the lack of co-ligand currently yields the highest polarization for this biomolecule, rationalizing the choice of our experimental conditions [50,61].

### 2.2. “Canonical” SABRE-SHEATH using static microtesla fields

**Scheme 1.**
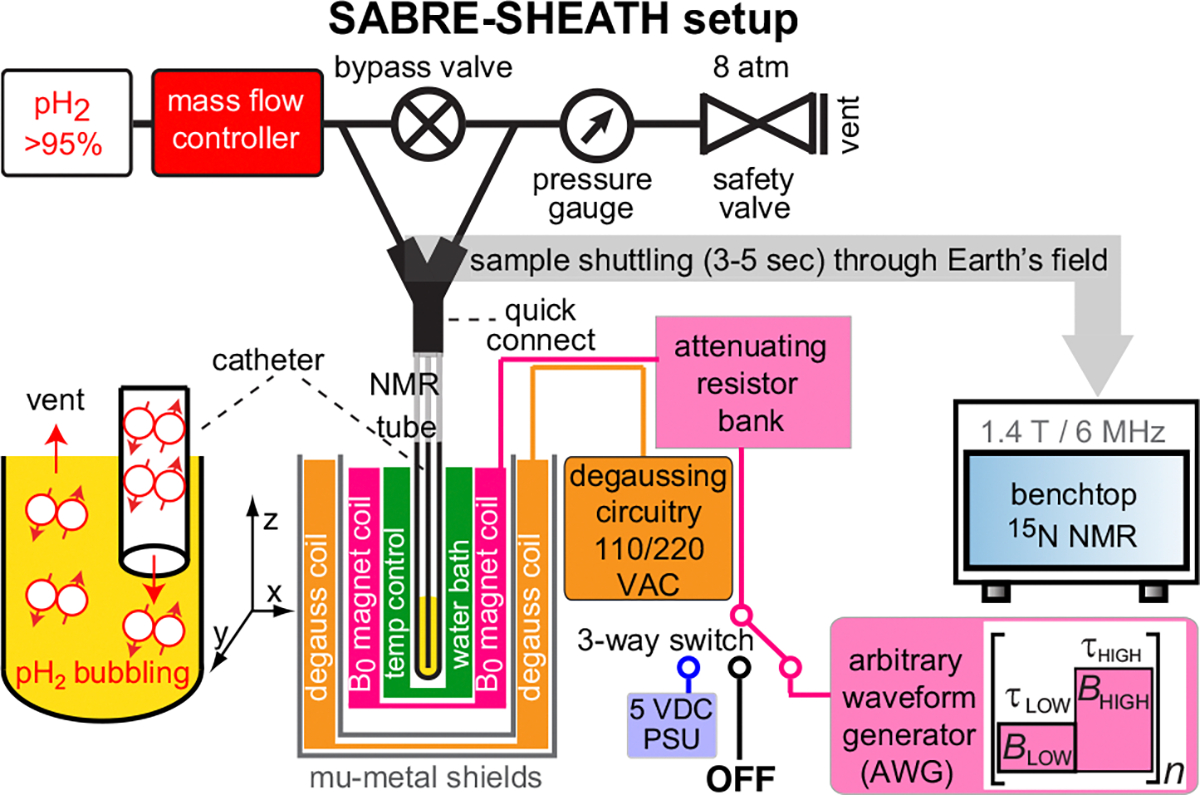
Schematic of experimental setup (*B*_0_ field inside the shields is applied along the *z*-direction; note that residual *x-y* static magnetic fields may exist in the shields [60]).

The hyperpolarization by SABRE-SHEATH was performed using an integrated setup described elsewhere and schematically shown in Scheme 1 [60]. Briefly, this device consists of a two-layered mu-metal shield (ZG-206, Magnetic Shield, Corp., Bensenville, IL, USA) to attenuate the Earth’s magnetic field to <40 nT residual field [62]. A fixed field of ~0.80 μT inside the shield was generated using a compensated solenoid powered by a DC power source [60]. This solenoid coil has 90 % homogeneity over its 7″ length to make sure the entire HP sample experiences a relatively homogenous microtesla field in the shields. All microtesla fields were generated via this solenoid coil, which employed a 5 VDC power supply equipped with a decade resistor bank [60]. For SABRE-SHEATH hyperpolarization, each sample was then bubbled with pH_2_ (95–98 % parahydrogen state [63]) at ~8 atm total pressure and 70 sccm flow at room temperature (except for temperature sweep studies) using a digital mass flow controller, Scheme 1 [62]. A three-axis fluxgate magnetometer (Mag-03, Bartington, Witney, U.K.) was used to measure and confirm all fields in all directions with a particular emphasis on the *z*-direction.

### 2.3. Pulsed SABRE-SHEATH using rectangular pulses

For the pulsed microtesla experiments, an arbitrary waveform generator (AWG) waveform was employed as a source of the pulses, which were attenuated by the same resistor connected to the same solenoid coil as discussed above. To calibrate the pulsed fields, a test waveform was initially loaded with a period of 4 s and a duty cycle of 2 s onto the AWG), and the field inside the shield was monitored with the magnetometer to confirm that the desired fields were indeed generated by the solenoid coil inside the shield. This step was necessary because the pulsed SABRE-SHEATH sequence employed millisecond-long pulse durations, which were captured by the magnetometer as an average field, and thus, preventing precise calibration of the individual pulses. The duration of the pulses was confirmed by the oscilloscope. Once the pulse amplitudes and duration were confirmed, the activated sample was placed inside the solenoid in the shield, and the pulse sequence was turned on simultaneously with the pH_2_ bubbling. Each sample was then bubbled with pH_2_ at ~8 atm total pressure and 70 sccm flow rate at room temperature using a digital mass flow controller, Scheme 1. After bubbling pH_2_ for 60 s (except for $P_{15N}$ build-up studies, where the bubbling time was varied), the bubbling was stopped, and the sample was allowed to settle for a second and then manually transferred (3–5 s) to a 1.4 T bench-top NMR spectrometer (NMR Pro60, Nanalysis). The overall sequence is schematically shown in the inset of the AWG block, and consists of two blocks of low magnetic field (*B*_LOW_) with duration of τ_LOW_ followed by a higher field pulse (*B*_HIGH_) with duration of τ_HIGH_. These two blocks were loaded as a sequence and repeated indefinitely in the AWG settings.

### 2.4. ^15^N NMR data acquisition for ^15^N polarization build-up measurements

All build-up curves were obtained by keeping all experimental parameters the same and then varying only the pH_2_ bubbling time. To obtain each data point (*x,y*), pH_2_ was bubbled into the solution for *x* seconds inside the shield and then transferred for detection in the 1.4 T NMR spectrometer for acquisition to calculate polarization value *y*. A 90° excitation pulse was employed for ^15^N signal detection. Next, the sample was returned back to the shield, where it was equilibrated for at least 2 mins prior to the next experiment with a different duration of pH_2_ bubbling. The $P_{15N}$ / time arrays’ values were fitted employing a mono-exponential build-up function using Origin Pro software to obtain a value for the exponential build-up constant and the associated error bars.

### 2.5. ^15^N NMR data acquisition for static in-shield ^15^N polarization T_1_ decay measurements

The polarization was allowed to build-up for 60 s during pH_2_ bubbling for each measurement using the optimal field of ~0.8 μT. The pH_2_ bubbling was then stopped by opening the bypass valve and the field was set to the desired static field using an attenuating resistor bank, Scheme 1. Next, the sample was allowed to stay in the shield for ^15^N polarization to decay for a given number of seconds before being rapidly transferred to high field (1.4 T) for detection. One data point was acquired for every experiment. Upon completion of the detection, the sample was returned back to the shield, where it was equilibrated for at least 2 mins prior to the subsequent experiment. Multiple (*x,y*) data points were collected as described above, and then fitted employing a mono-exponential decay function using Origin Pro software to obtain a value for the exponential decay constant and the associated error bars.

### 2.6. ^15^N NMR data acquisition for pulsed in-shield ^15^N polarization T_1_ decay measurements

These experiments were performed similarly to the ones described in the above section with a small difference: The polarization build-up was performed by applying the shield’s pulse sequence to run continuously during pH_2_ bubbling for 60 s. Next, the pH_2_ bubbling was stopped, and the sample was left to sit in the shield for $P_{15N}$ decay while the shield’s pulse sequence was allowed to run during that decay process.

### 2.7. ^15^N NMR data acquisition for ^15^N polarization T_1_ decay in the Earth’s field

These experiments were performed similar to the $P_{15N}$ *T*_1_ decay experiments in the static magnetic fields as discussed above, with one small modification: upon completion of $P_{15N}$ build-up in the shield at ~0.8 μT, pH_2_ bubbling was stopped, and the sample was quickly transferred out of the shield and into the Earth’s field. $P_{15N}$ decay in this case occurred in the Earth’s field versus the field of the shield. One data point was obtained from each experiment, similarly to the decay studies described above.

### 2.8. ^15^N NMR data acquisition for ^15^N polarization T_1_ decay in 1.4 T field

These studies were performed by first hyperpolarizing the sample using pH_2_ bubbling in the shield for 60 s at ~0.8 μT and room temperature. Next, pH_2_ bubbling was stopped and the sample was quickly transferred to the 1.4 T NMR spectrometer for detection. Here, multiple ^15^N acquisitions were performed using a 10° excitation pulse applied every 1 min. The $P_{15N}$ decay data was fitted to a mono-exponential decay function as discussed above. The effect of the pulse excitation was not taken into account for *T*_1_ computation due to concerns of sample convection and residual motion during one-minute long intervals between acquisitions.

### 2.9. ^15^N polarization quantification using a benchtop 1.4 T NMR spectrometer

All ^15^N spectra from HP samples were recorded at 20 mM concentration ($C_{\text{HP}}$) of [^15^N]metronidazole (e.g., Fig. 1b): this concentration was employed for $P_{15N}$ computation using Eq. (1). To compute each signal enhancement value ($\varepsilon_{15\;N}$), the detected signal from a given HP sample ($S_{\text{HP}}$) was referenced against the ^15^N signal ($S_{\text{REF}}$) from a thermally polarized sample (neat [^15^N]pyridine, $C_{\mathrm{REF}}=12.4\;\text{M}$). Thermally polarized 20 mM [^15^N]metronidazole cannot be readily utilized for signal referencing due to low concentration, that would require signal averaging, which is hardly possible with ultra-long ^15^N *T*_1_ values at 1.4 T. Since the reference sample was loaded in a regular-wall 5-mm NMR tube (without Teflon catheter for pH_2_ bubbling), an additional correction factor was applied to accommodate for the difference in the effective solution cross-sectional area of the NMR tube ($A_{\text{REF}}/A_{\text{HP}}$). This correction factor was measured experimentally (1.52), yielding the relation provided below (Eq. (1)). The detection protocol for HP and thermally polarized samples was the same, except that thermally polarized sample required 64 signal averages recorded with a repetition time of 10 min.

(1) $$\varepsilon_{15N}=\frac{S_{HP}}{S_{REF}}\times \frac{C_{REF}}{C_{HP}}\times \frac{A_{REF}}{A_{HP}}=\frac{S_{HP}}{S_{REF}}\times \frac{C_{REF}}{C_{HP}}\times 1.52$$

The $P_{15N}$ value was computed according to Eq. (2), using the ^15^N thermal polarization level at 1.4 T and 298 K ($P_{\mathrm{therm}}=4.86\times 10^{-5}\;\%$).

(2) $$P_{15N}=\varepsilon_{15N}\times P_{\mathrm{therm}}$$

## 3. Results and discussion

### 3.1. Overall rationale

Pulsed SABRE-SHEATH employing pulses of magnetic field in contrast to the static SABRE-SHEATH (where the magnetic field kept constant throughout the polarization transfer process) can generate coherent spin evolution, which can in principle be a more efficient approach [38]. The relevant polarization-transfer interaction (^2^*J*-coupling interaction ^2^*J*_H-H’_ and ^2^*J*_HN_) are on the order of 7–30 Hz, thus, the anticipated approximate durations of the pulses are on the order of 1–10 ms (in the first approximation (*J*_effective_)^−1^) that would lead to creation of coherent polarization transfer pathways [38]. In contrast, the static-field SABRE-SHEATH in an incoherent evolution process.

### 3.2. ^15^N polarization dynamics

**Fig. 2.**
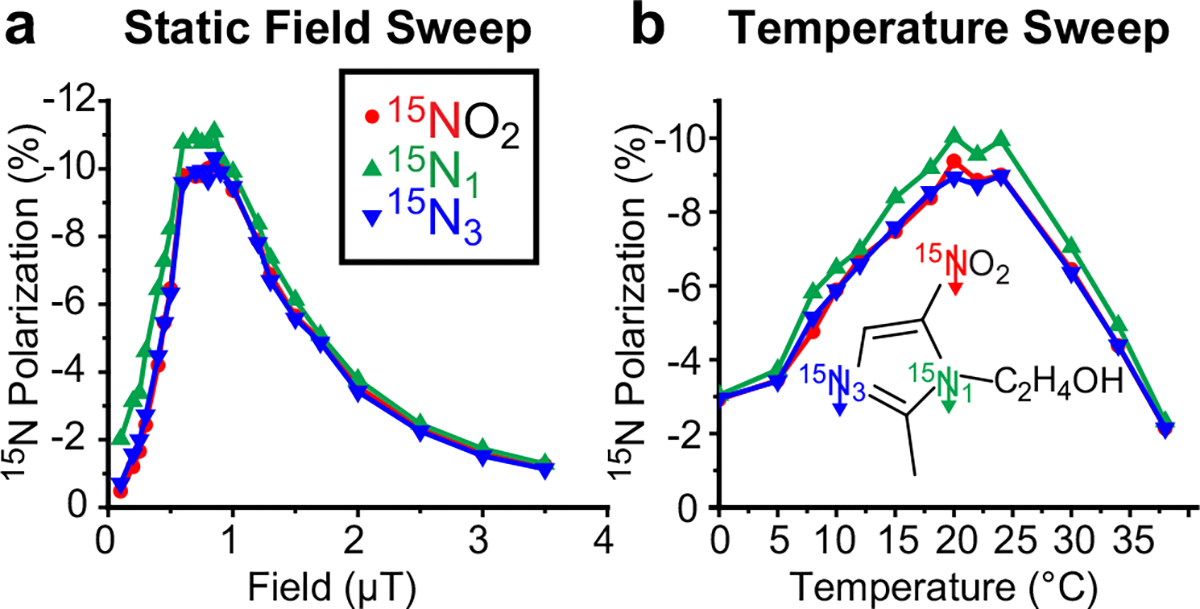
a) Dependence of ^15^N_3_ polarization ($P_{15N}$) of [^15^N_3_]metronidazole on the static field strength at fixed (room) temperature. b) Dependence of ^15^N polarization of [^15^N_3_]metronidazole on temperature at a fixed static field of ~0.8 μT. All experiments were performed with ~20 mM [^15^N_3_]metronidazole and 2 mM IrCl(COD)(IMes) pre-catalyst in CD_3_OD. Note the color-coding for three ^15^N sites. Note the negative sign of ^15^N polarization refers to the opposite sign of gained ^15^N polarization with respect to the thermally induced equilibrium ^15^N polarization at 1.4 T NMR spectrometer.

We first performed a static field sweep experiment using the conventional SABRE-SHEATH method to locate the optimal conditions for the static field and temperature, see Fig. 2a and 2b respectively. This baseline experiment is performed to have control measurements for the $P_{15N}$ and relaxation dynamics for comparison with pulsed SABRE-SHEATH. Overall, the optimum static field for this PTC was found to be ~0.8 μT, and optimum temperature around 17–18 °C (corresponding to room temperature condition), in line with previous studies [64].

**Fig. 3.**
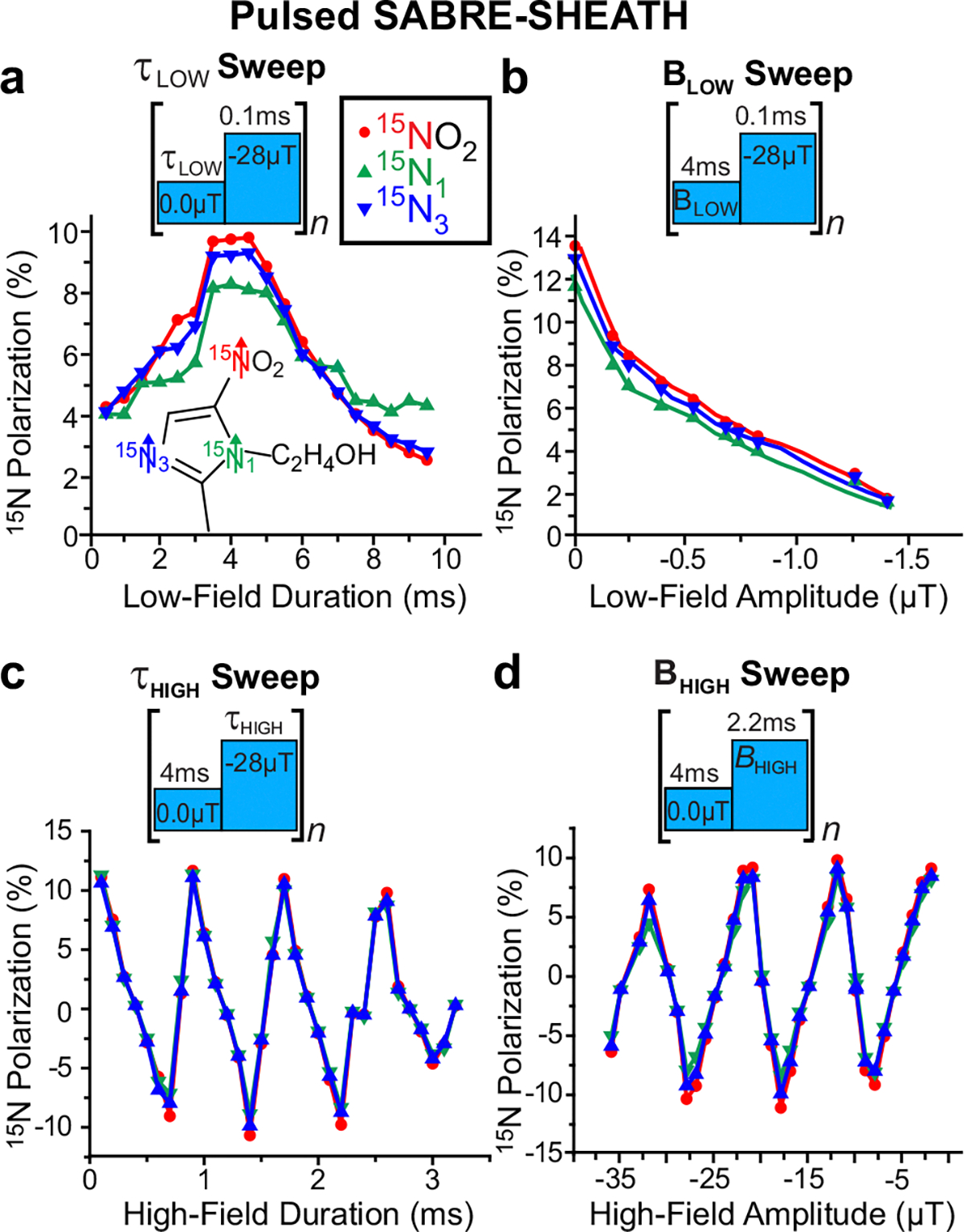
Pulsed SABRE-SHEATH: dependence of ^15^N polarization of the three ^15^N spins of [^15^N_3_]metronidazole on the duration of the high-field period, τ_HIGH_ (a); the duration of the low-field period, τ_LOW_ (b); the low-field pulse amplitude, *B*_LOW_ (c); and high-field pulse amplitude, *B*_HIGH_ (d). All experiments were performed with ~20 mM [^15^N_3_]metronidazole and 2 mM IrCl(COD)(IMes) pre-catalyst in CD_3_OD at room temperature. The solid lines between points are meant only to guide the eye. Figure S1 shows a reproducibility study of these results with the simulations’ overlays.

Next, our studies progressed to the pulsed SABRE-SHEATH: A low-field pulse duration sweep (τ_LOW_) was performed by arbitrarily setting the high-field pulse duration (τ_HIGH_) to 0.1 ms, the high field amplitude (*B*_HIGH_) to −28 μT, and the low field amplitude (*B*_LOW_) to ~0.00 μT; the value for τ_LOW_ was then systematically varied. It was found that the optimum low-field duration of our sequence was around 4 ms, Fig. 3a (and also in Figure S1a). This value is in agreement with the result previously obtained for [^15^N]acetonitrile [34], which is rationalized by the similarity of the spin-spin couplings in the corresponding PTCs formed in both cases [34]. We also anticipate that a wide range of ^15^N-contatining heterocycles amenable to simple ^15^N enrichment [65] will likely have similar spin-spin coupling networks within the corresponding PTCs, thus making 4 ms a relatively universal value τ_LOW_—which should help make sequence optimization for other substrates relatively straightforward.

As we have seen in systems studied previously, such as [^15^N]acetonitrile [37], the values on the order of a few ms for τ_LOW_ and τ_HIGH_ on the order of 0.1 ms are good starting points for the exploration of this new approach for [^15^N_3_]metronidazole hyperpolarization under pulsed-SABRE-SHEATH studied here. Thus, having established the optimum τ_LOW_ value, we then proceeded further to perform a *B*_LOW_ sweep by fixing the values of *B*_HIGH_, τ_HIGH_, and τ_LOW_ at −28 μT, 0.1 ms, and 4 ms, respectively. Interestingly, the highest polarization level was found near a low-field amplitude of 0.00 μT, and $P_{15N}$ tapered off as the low-field pulse field strength progressively decreased to −1.5 μT, Fig. 3b (and also Figure S1b). This observation was also in is agreement with the previous studies for [^15^N]acetonitrile spin system [34]. Further in the optimization process was a sweep through the high-field pulse duration while fixing the values for the high-field amplitude, the low-field amplitude, and the low-field duration at −28μT, 0.0 μT, and 4 ms respectively (i.e., using the optimized values of *B*_LOW_ and τ_LOW_). We see periodic $P_{15N}$ oscillation as we sweep across the range, with multiple maxima occurring close to every 2·π·n rotations [34], Fig. 3c (and also Figure S1c). These maxima together with the minima occur when the high-field pulse duration induces an angular rotation θ_HIGH_ = 2·π·n s [34].

**Fig. 4.**
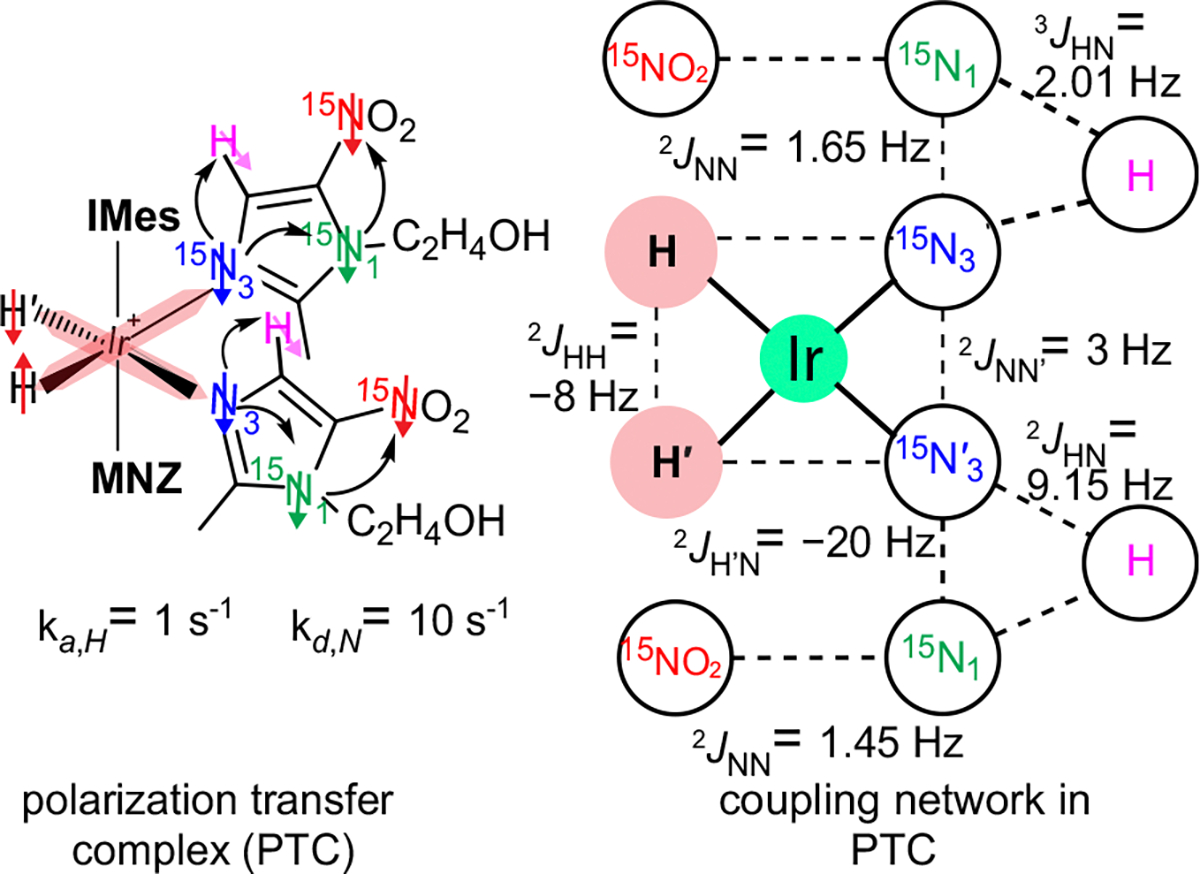
Schematic of the spin system and relevant parameters employed in the numerical simulations. All parameters used in the simulations were either defined (JH-H′2=−8Hz, k_a_,*H* = 1 s^−1^), set to the experimental values (magnetic field sequence, relative concentrations of ligand and catalyst), or varied to fit the experimental data (^3^*J*_N–*H*_, k_d_,N).

In the final step of the optimization, the optimal values of *B*_LOW_, τ_HIGH_ and τ_LOW_ were employed, and a high-field pulse magnitude sweep was performed—also demonstrating a periodic pattern behavior (Fig. 3d and also Figure S1c). In Figure S1, the individual symbols (color-coded circles and triangles) represent the experimental data, and the solid color-coded lines correspond to the simulations (except for the data presented in Figure S1a, where simulations were not performed); here, the simulations were performed using a similar approach to that presented recently for [1–^13^C]pyruvate [39], but using the parameters of the spin system shown in Fig. 4. The reader is also referred to other recent works related to pulsed SABRE and the detailed theoretical description [36,66]. The agreement between the theoretical simulations and the experimental data was good, Figure S1, highlighting the overall good understanding of the polarization transfer process in the previously developed theory [34].

The position of the first maximum on the periodic curve shown in Fig. 3c and Figure S1c can be readily rationalized as follows: the average field experienced by the ^15^N spins is approximately equal to the optimal field value of the static SABRE-SHEATH (~0.8 μT). However, all other maxima can no longer be rationalized using this averaged-field approach, and they are the results of non-intuitive polarization dynamics [34,37]. Moreover, the duration of the low-field pulse is crucial, and the optimum is dependent on the exchange rate of the substrate and pH_2_ on PTC. If the duration is too short or too long compared to the exchange rate of the substrate on the polarization transfer catalyst, then the complex will mostly experience either only the low field or the high field throughout its lifetime, which does not translate to efficient polarization transfer [34,37].

The presented results are rationalized by assuming that the ^15^N_3_ nucleus is the only binding nucleus to the PTC, and for all intents and purposes related to polarization transfer from the pH_2_-derived hydrides. The ^15^N_1_ and ^15^NO_2_ nuclei were neglected in the treatment of the pulse sequence’s effect on polarization transfer. This assumption is reasonable since there are no appreciable spin-spin couplings between pH_2_-derived hydrides and ^15^N_1_ and ^15^NO_2_ sites. The build-up of ^15^N polarization on ^15^N_1_ and ^15^NO_2_ nuclei is therefore due to spin-relayed polarization transfer, which has been studied previously for the static SABRE-SHEATH case [49,53,67]. In this model, the polarization of ^15^N_3_ site is effectively shared with the spin-spin coupled ^15^N_1_ site (via ^2^*J*_NN_ = 1.65 Hz [49,53]), which then establishes its own polarization transfer relay with the ^15^NO_2_ site via ^2^*J*_NN_ of 1.45 Hz [49,53]. Notably, $P_{15N}$ values of all three sites are nearly the same, even at high values of τ_HIGH_ (Fig. 3c) and of *B*_LOW_ (Fig. 3d). These results clearly indicate that the spin-relayed polarization transfer remains highly efficient over a broad range of microtesla fields, representing an important finding that supports the broad applicability of pulsed SABRE-SHEATH for hyperpolarizing multi-labeled substrates. In particular, the spin-relayed polarization transfer at fields substantially above one microtesla represents a promising path for hyperpolarization of a wide range of biomolecules beyond ^15^N-^15^N spin-spin coupled sites in drugs such as metronidazole: we envision that similar schemes may also be established between ^13^C-^13^C pairs in biomolecules.

### 3.3. Limitations of the studies and the comparative efficiency of pulsed versus static SABRE-SHEATH

Unlike static SABRE-SHEATH, which is performed at the same field, requiring virtually no additional optimization besides the field and the temperature, Fig. 2, the experiments summarized in Fig. 3 and Figure S1 for pulsed SABRE-SHEATH were performed over the course of several hours. During this long period of time, the catalyst performance changes (see SI for details, e.g., Figure S2), thus, adding variability to the absolute $P_{15N}$ levels throughout long experiments (and while the position of the maxima and minima is not affected, the actual signal intensities and $P_{15N}$ values are being affected). The highest $P_{15N}$ observed via pulsed SABRE-SHEATH was 18.5 % (Fig. 1 and Figure S1). To address this limitation of sample deterioration over multi-hour period, we have performed additional back-to-back comparative studies using pulsed and static SABRE-SHEATH variants with minimal amount of time between them (~5 mins, see Figure S3 for details). These additional experimental series revealed that the pulsed SABRE-SHEATH method yielded (1.32 ± 0.14)-fold improvements in $P_{15N}$ compared to static variant, which is similar to 1.3-fold gain observed in previous [^15^N] acetonitrile study [38]. It should also be additionally noted that the maximum obtained $P_{15N}$ in the studies reported here was 18.5 % for the pulsed technique; utilizing the (1.32 ± 0.14)-fold improvement factor, a $P_{15N}$ of 14 ± 2 % is anticipated for the static-field SABRE-SHEATH technique under otherwise the same conditions, which matches closely a previously reported value of $P_{15N}$ of 15 % for [^15^N_3_]metronidazole [49,53].

### 3.4. ^15^N relaxation dynamics

In the microtesla field range, especially close to near zero field (below 1 microtesla), the dephasing effects may lead to substantially faster depolarization of the HP state [39], so strictly speaking relaxation at such low fields may not be called *T*_1_ decay of HP state (and therefore we denote the ^15^N polarization mono-exponential decay constants at such low fields as *T*_d_ to emphasize the fact that the decay is due to more than just *T*_1_ relaxation processes). At the same time, the reader is reminded that dephasing may not necessarily be dominant depolarization mechanism: the degree of dephasing contribution depends on the experimental setup and the ratio of the residual static field and imperfections of the experimental setup.

**Fig. 5.**
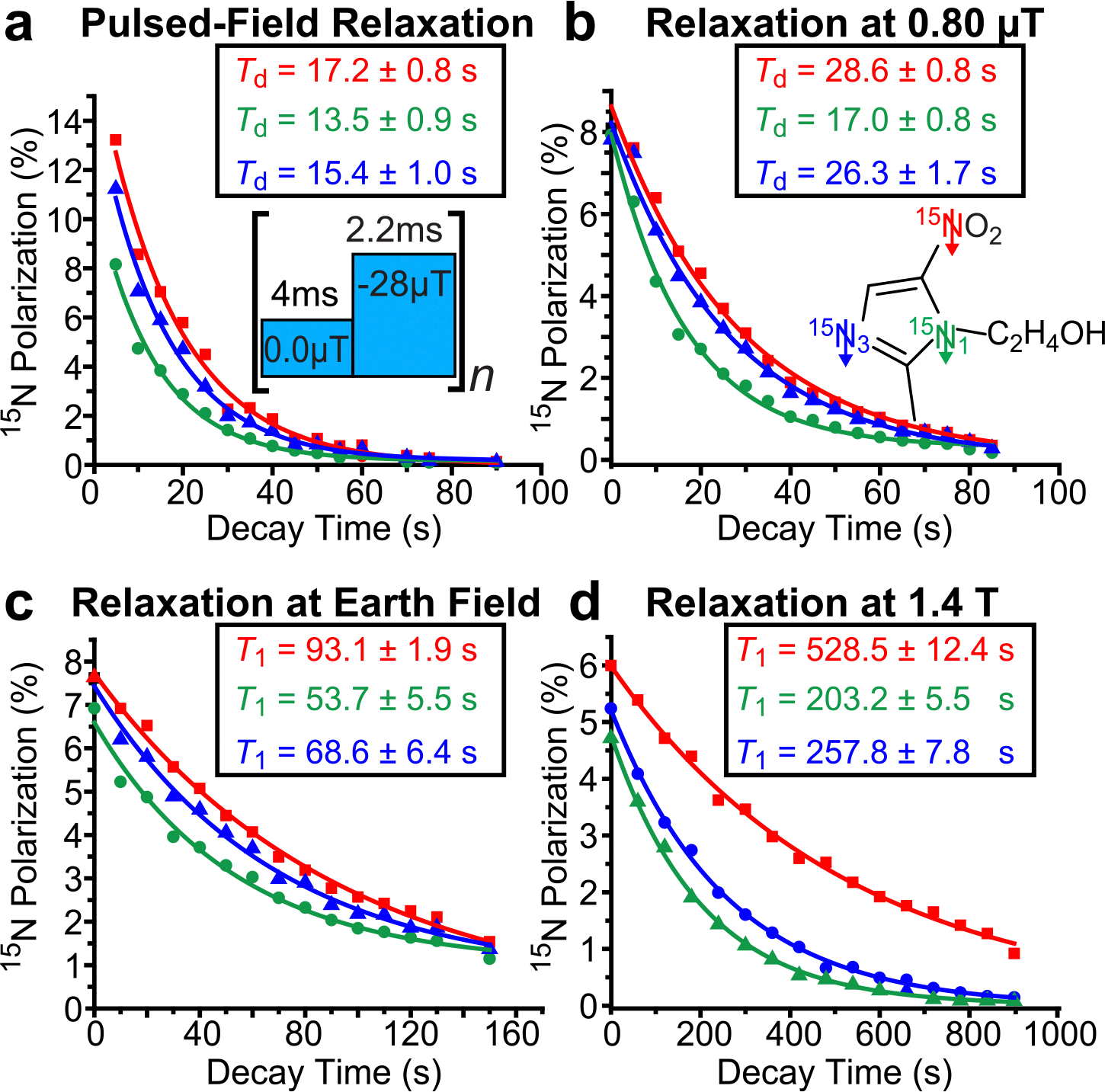
a) ^15^N *T*_1_ polarization decay of [^15^N_3_]metronidazole during application of optimized SABRE-SHEATH pulses. b) ^15^N polarization decay of [^15^N_3_]metronidazole at 0.80 μT static field. c) ^15^N *T*_1_ polarization decay of [^15^N_3_]metronidazole at the Earth’s field (ca. 50 μT), and d) ^15^N *T*_1_ polarization decay of [^15^N_3_] metronidazole at 1.4 T All experiments were performed with ~20 mM [^15^N_3_]metronidazole and 2 mM IrCl(COD)(IMes) pre-catalyst in CD_3_OD at room temperature. $P_{15N}$ values are listed in the magnitude mode.

**Fig. 6.**
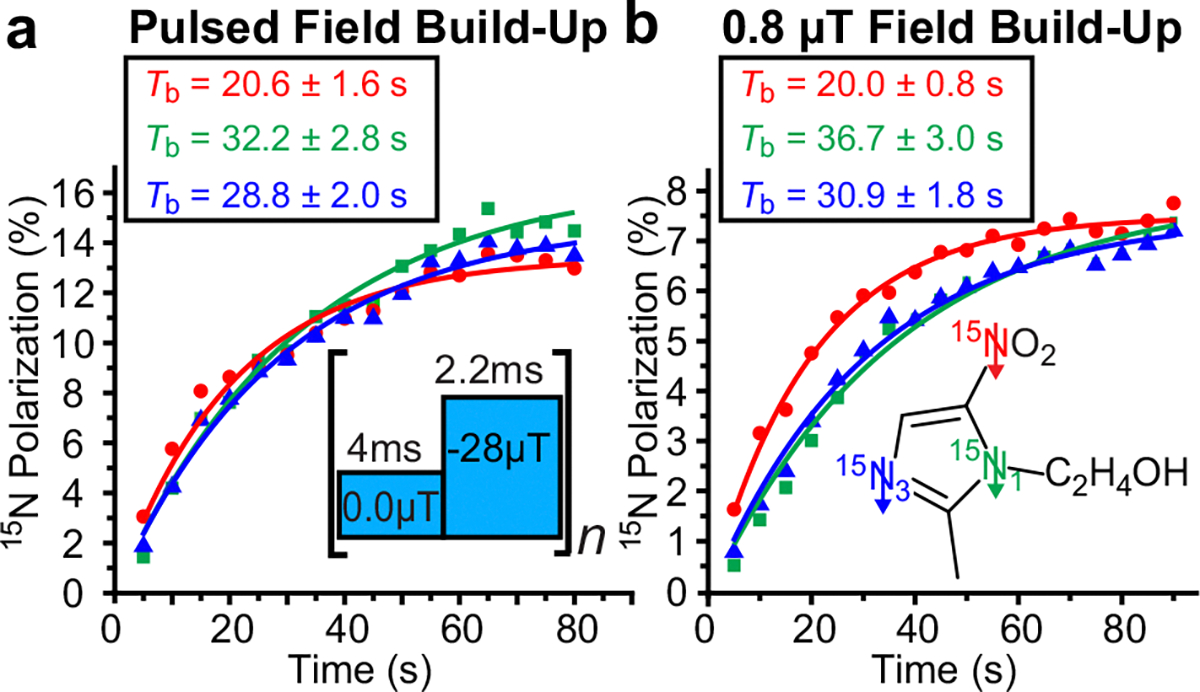
a) ^15^N_3_ polarization build-up of [^15^N_3_]metronidazole via pulsed SABRE-SHEATH using τ_LOW_=4 ms; τ_HIGH_=2.2 ms; |*B*_LOW_|<40 nT, *B*_HIGH_= −28 μT. b) ^15^N_3_ polarization build-up of [^15^N_3_]metronidazole via conventional static-field SABRE-SHEATH at ~0.80 μT. All experiments were performed with ~20 mM [^15^N_3_]metronidazole and 2 mM IrCl(COD)(IMes) pre-catalyst in CD_3_OD at room temperature. $P_{15N}$ values are listed in the magnitude mode.

An examination of the ^15^N decay data at the pulsed microtesla fields shows that $P_{15N}$ decay is in fact faster during the variable pulse-field conditions (Fig. 5a) than that at static 0.8 μT field (Fig. 5b) for all three ^15^N sites. This observation is rather unexpected because of the following reasons: Previous ^13^C decay studies at similar magnetic fields and using similar pulse schemes revealed that even a short pulse duration (0.1 ms) at high pulse field strength amplitude (28 μT) is capable of fully rephasing any dephasing that the nuclear spins may experience at near zero field, even if they spend a longer time there (e.g., 6 ms vs. 4 ms studied in Fig. 5a) [39]: here the duration of the high-field pulse was substantially longer: 2.8 ms (and thus, a full rephasing is anticipated with virtually negligible effects on effective ^15^N *T*_d_). Moreover, the average field experienced by the spins during pulsing is 10 μT, i.e., an order of magnitude greater than the 0.8 μT field employed for the static SABRE-SHEATH experiments. As the microtesla field increases, the ^15^N *T*_d_ and *T*_1_ values are anticipated to increase too—for example, the ^15^N *T*_1_ values at the Earth’s field (ca. 50 μT, Fig. 5c) reveal relaxation values that are approximately 3 times greater than those at 0.8 μT. A likely explanation is that the pulsed SABRE-SHEATH approach enables other efficient pathways for polarization transfer from ^15^N sites to proton nuclei in the molecular structure of [^15^N_3_]metronidazole. As protons have inherently shorter *T*_1_ values in general (due to their larger magnetic moment), they lose the gained HP state faster, effectively acting as polarization sinks. This is not a fundamental limitation of the pulsed SABRE-SHEATH approach in general as potentially “smarter” approaches can be potentially developed that could offer more selective transfer of the polarization. The ^15^N polarization build-up rates were unremarkable: ^15^N *T*_b_ values were only slightly smaller for the pulsed approach (Fig. 6a) versus those for static SABRE-SHEATH (Fig. 6b), albeit within the uncertainty range of our measurements. Faster build-up values were indeed anticipated for the pulsed method due to more efficient ^15^N decay [68]. However, this is not the case: substantially higher $P_{15N}$ are achieved for all three ^15^N sites for the pulsed method versus static approach on the same sample, Fig. 6, despite the less favorable “apparent” relaxation dynamics. We reconcile these findings as follows: even though the $P_{15N}$ decay is less favorable for the pulsed scheme employed, the pulsed approach offers more-efficient polarization transfer, such that it still achieves a slightly higher $P_{15N}$ values overall.

**Fig. 7.**
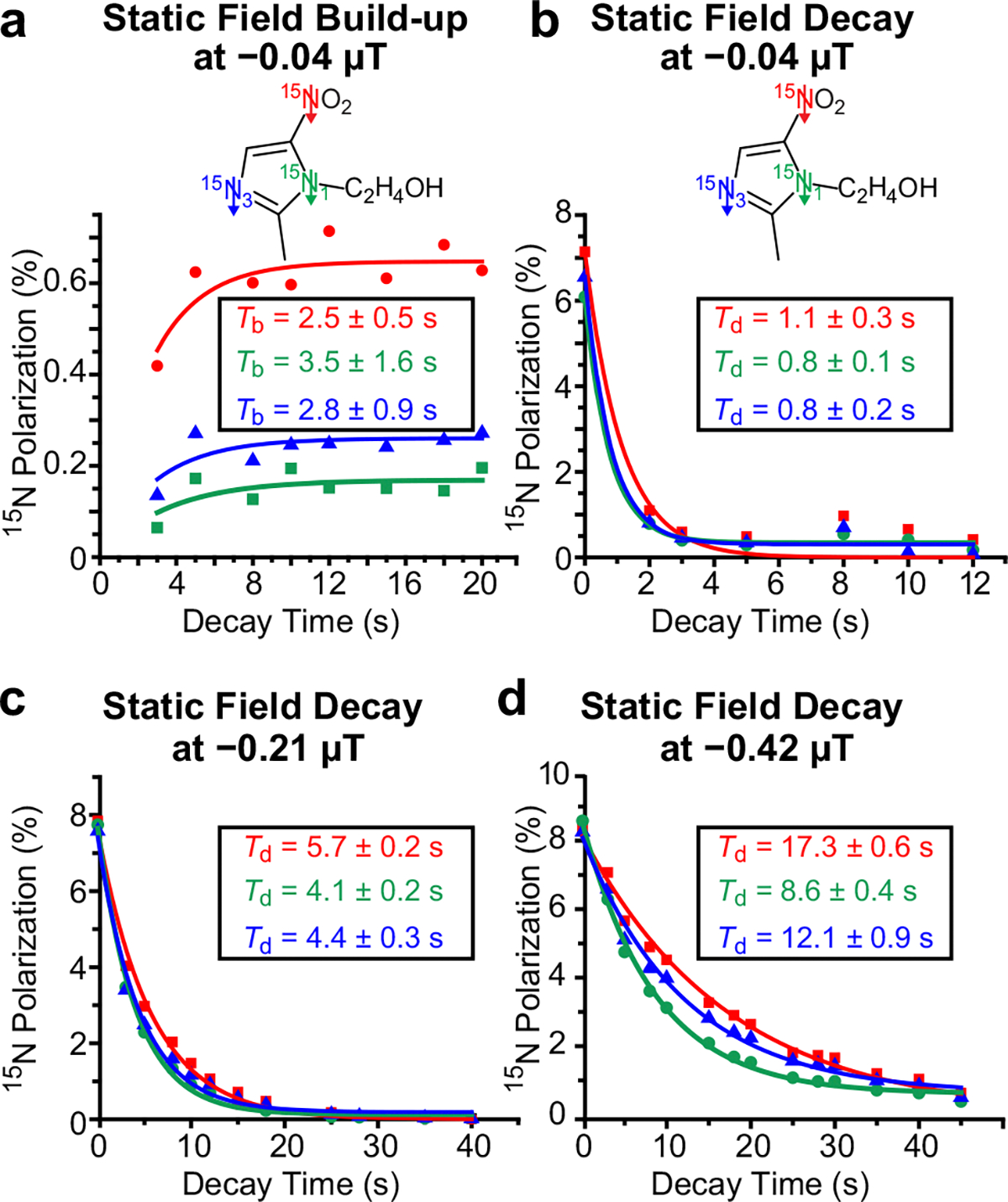
a) ^15^N *T*_1_ polarization build-up on [^15^N_3_]metronidazole at near zero field of −0.04 μT. b) ^15^N *T*_1_ polarization decay of [^15^N_3_]metronidazole due to polarization dephasing associated with the shields’ residual magnetization in the x-y plane at near zero field (−0.04 μT). c) ^15^N *T*_1_ polarization decay of [^15^N_3_]metronidazole due to polarization dephasing associated with the shields’ residual magnetization in the x-y plane at −0.21 μT. d) ^15^N *T*_1_ polarization decay of [^15^N_3_]metronidazole due to polarization dephasing associated with the shields’ residual magnetization in the x-y plane at −0.42 μT. $P_{15N}$ values are listed in the magnitude mode.

Additional control studies were performed, and they are reported in Fig. 7. As shown in Fig. 3a, the sample spends most of the time at near zero field value (4 ms vs. 0.1 ms at an optimum value). The control build-up studies (Fig. 7a) revealed fast build-up rates with low $P_{15N}$ values obtained in this process, thus ruling out the possibility of otherwise efficient $P_{15N}$ build-up at near zero field on its own. The fast build-up rates at near zero field are rationalized by efficient ^15^N polarization decay for all three ^15^N sites due to dephasing that may be caused by magnetic field inhomogeneities and residual fields orthogonal to the field created by our solenoid, Fig. 7b [39]. This notion is supported by the previous ^13^C studies with [1–^13^C]pyruvate [39] and additionally by the ^15^N decay data at −0.21 μT (Fig. 7c) and −0.42 μT (Fig. 7d). As the in-shield field increases, the effect of the spin dephasing (due to inhomogeneities and other residual fields) is reduced, which is revealed by the stepwise increase in the ^15^N *T*_d_ values from −0.04 μT (Fig. 7c) to −0.21 μT (Fig. 7c) to −0.42 μT (Fig. 7d) to 0.80 μT (Fig. 5b).

An additional control experiment was also performed to measure ^15^N *T*_1_ relaxation values at 1.4 T (Fig. 5d), which indeed revealed very long ^15^N *T*_1_ values of up to 9 mins—in agreement with previous 1.4 T relaxation studies [49,53].

## 4. Conclusion

We have demonstrated efficient hyperpolarization of [^15^N_3_]metronidazole by pulsed SABRE-SHEATH method. ^15^N polarization values of up to 18.5 % are obtained in 80 s at room temperature with this method. This value corresponds to the overall $P_{15N}$ improvement by a factor of 1.32 ± 0.14 over conventional SABRE-SHEATH. Based on the relaxation dynamics results, we hypothesize that the pulsed SABRE-SHEATH creates closer/more efficient contact and stronger interactions between pH_2_-derived hydrides and ^15^N which leads to (i) more efficient $P_{15N}$ build-up during pH_2_ bubbling, but also to (ii) more efficient $P_{15N}$ decay when the pH_2_ bubbling is turned off. We also hypothesize that the faster-than-anticipated $P_{15N}$ decay of the pulsed approach is due to more efficient ^15^N polarization transfer to intramolecular proton spins, which may effectively act as relaxation sinks. These protons include the hydrides, when the bubbling is turned off. The reported result is also significant because this is the first demonstration of the pulsed SABRE-SHEATH that has been successfully applied to the molecule containing multiple heteronuclei that can act as HP state carriers for in vivo applications [48]. Moreover, this is the first report (to the best of our knowledge) of successfully employing a pulsed SABRE-SHEATH approach to a ^15^N-labeled biologically relevant molecule. Furthermore, this high polarization level together with the high-field relaxation ^15^N *T*_1_ constant of up to 9 min (at a clinically relevant 1.4 T field) makes HP [^15^N_3_]metronidazole a potentially useful molecular imaging probe [50]. The reported levels of ^15^N polarization indeed surpass those obtained by the d-DNP method by >3 fold [48]. It is our anticipation that further improvement in the pulsed approach may substantially increase polarization for other ^15^N-containing drugs and biologically relevant molecules [54–58].

## Supplementary Material

1

Supplementary material associated with this article can be found, in the online version, at doi:10.1016/j.jmro.2025.100208.

## Data Availability

All raw spectroscopic data is freely available via https://data.mendeley.com/datasets/b6vh3n4y44/1 under doi: 10.17632/b6vh3n4y44.1.
