# Supplementary material for: Efficient 15N hyperpolarization of [15N3]metronidazole antibiotic via spin-relayed pulsed SABRE-SHEATH
Source: J Magn Reson Open. Author manuscript; Available in PMC 2026 Jun 30. (PMC13313728; doi:10.1016/j.jmro.2025.100208)
Supplement: 1 [file NIHMS2180280-supplement-1.pdf]

SUPPORTING INFORMATION FOR

Efficient ^{15}N Hyperpolarization of [$^{15}N_3$]Metronidazole Antibiotic via Spin-Relayed Pulsed SABRE-SHEATH

Shiraz Nantogma,[a] Shannon L. Eriksson,[b,e] Thomas Theis,[c] Warren S. Warren,[b,d,e,f] Boyd M. Goodson,[g] and Eduard Y. Chekmenev*[a]

[a]Department of Chemistry, Integrative Biosciences (IBio), Karmanos Cancer Institute (KCI), Wayne State University, Detroit, Michigan 48202, United States
[b]Department of Chemistry, [d]Biomedical Engineering, and Radiology, [e]School of Medicine, [f]Department of Physics, Duke University, Durham, North Carolina 27708, United States
[c]Department of Chemistry, North Carolina State University, Raleigh, North Carolina, 27695-8204, United States
[g]School of Chemical & Biomolecular Sciences and Materials Technology Center, Southern Illinois University, Carbondale, Illinois 62901, United States

Table of Contents

1. Repeat studies of high field duration and high field amplitude sweeps S3

2. Activation of [$^{15}N_3$]metronidazole SABRE solutions at 15 sccm pH_2 flow rate S4

3. Systematic comparison of P_{15N} obtained via pulsed SABRE-SHEATH and static SABRE-SHEATH experiments S5

4. Example of P_{15N} calculations S7

1. Repeat studies of high field duration and high field amplitude sweeps

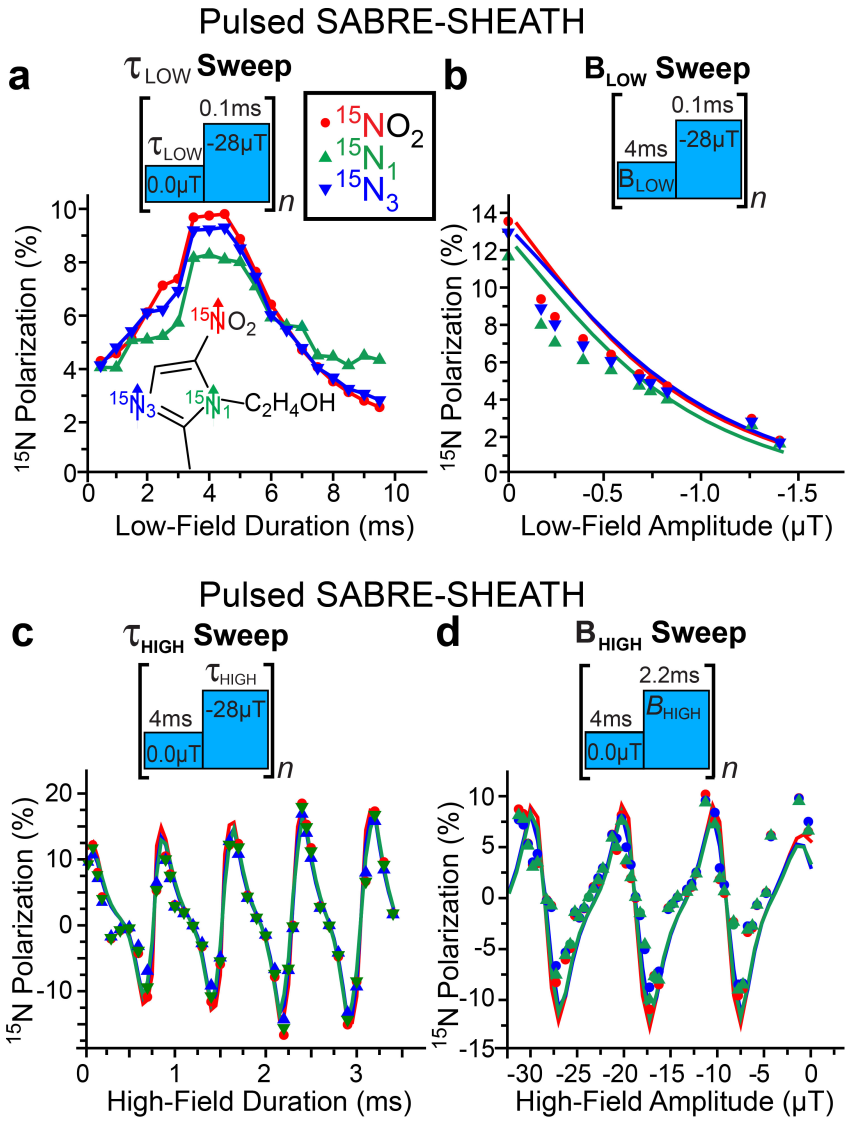

Figure S1. Pulsed SABRE-SHEATH: dependence of ^{15}N polarization of the three ^{15}N spins of [$^{15}N_3$]metronidazole on the duration of the high-field period τ_{HIGH} (a) the duration of the low-field period τ_{LOW} (b) the low-field pulse amplitude B_{LOW} (c) and high-field pulse amplitude B_{HIGH} (d). All experiments were performed with ~20 mM [$^{15}N_3$]metronidazole and 2 mM IrCl(COD)(IMes) pre-catalyst in CD_3OD at room temperature. Except for (a) all solid lines are simulations of the experimental data whereas in (a) they are only meant to guide the eye.

2. Activation of [$^{15}N_3$]metronidazole SABRE solutions at 15 sccm pH_2 flow rate

It is very important to take into consideration various experimental limitations that affect the results of these studies. Below are some important considerations:

i. Variations in sample concentration: Since it takes prohibitively long times to acquire a set of data to represent a trend such as the high field duration and high field amplitude sweeps, there is a boiling of the sample which leads to variation in the sample concentration over time. This in turn affects the polarization values. Moreover, it becomes difficult to obtain a complete dataset for a specific sample due to the sample loss. This limitation was unavoidable when recording data over 4-6 hours periods. However, the changes in metronidazole concentration were accounted for by measuring the sample height (and the corresponding sample volume reduction).

ii. Fluctuations in μT fields: Over time, both the low and high fields deviate from the preset values and as such this can shift the positions of the polarization maxima by a few micro- to milli-seconds. This potential issue was accounted for by degaussing the shield, and also by repeating the experiments, and ensuring the maxima and minima positions are reproducible.

iii. Sample activation and deactivation: the potency of the sample increases in the first 50 minutes followed by a steady decay of the maximum attainable P_{15N} level. This limitation of the study was unavoidable during the long multi-hour experiments. While the maxima and minima in the trends (e.g., those shown in **Figure 3**) do not change, the actual P_{15N} level were affected by this limitation. Therefore, the respective P_{15N} values of various experimental series should be treated with caution and should not be directly compared on a fully quantitative basis.

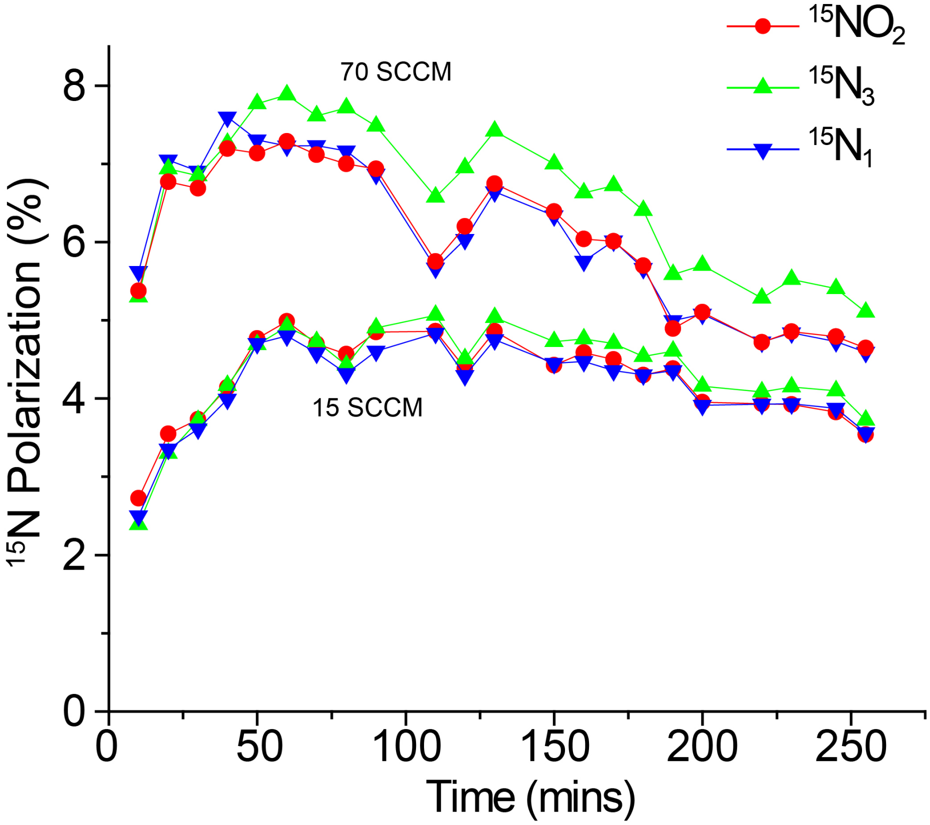

Figure S2. Activation of [$^{15}N_3$]metronidazole samples. Two trends are shown for the same sample the activation of which was monitored through the activation process. The activation was performed at 15 sccm pH_2 flow rate throughout the process. During this process data points were taken to measure P_{15N} throughout the process (in case when polarization was checked, it was checked using the buildup of polarization under both 15 sccm and 70 sccm pH_2 flow during polarization process). P_{15N} throughout is presented in the magnitude mode.

3. Systematic comparison of P_{15N} obtained via pulsed SABRE-SHEATH and static SABRE-SHEATH experiments

Unlike static SABRE-SHEATH, which is performed at the same field, requiring virtually no additional optimization besides the field and the temperature, the experiments summarized in **Figure 3** and **Figure S1** (reproducibility demonstration) for pulsed SABRE-SHEATH were performed over the course of several hours. During this long period of time (as discussed above), the catalyst performance changes, thus, adding variability to the absolute P_{15N} levels throughout long experiments (and while the position of the maxima and minima is not affected, the actual signal intensities and P_{15N} values are being affected). To address this limitation of sample deterioration over multi-hour period, we have performed additional back-to-back comparative studies using pulsed and static SABRE-SHEATH variants with minimal amount of time between them.

The results of these additional experimental series are shown in **Figure S3** and **Table S1**. Here, P_{15N} values were compared between matched series, and the ration of P_{15N} obtained via the pulsed SABRE-SHEATH method was compared to the P_{15N} values obtained via the static method. Standard deviations were also computed, yielding (1.32±0.14)-fold improvements in P_{15N} value for the pulsed-SABRE-SHEATH method compared to static variant.

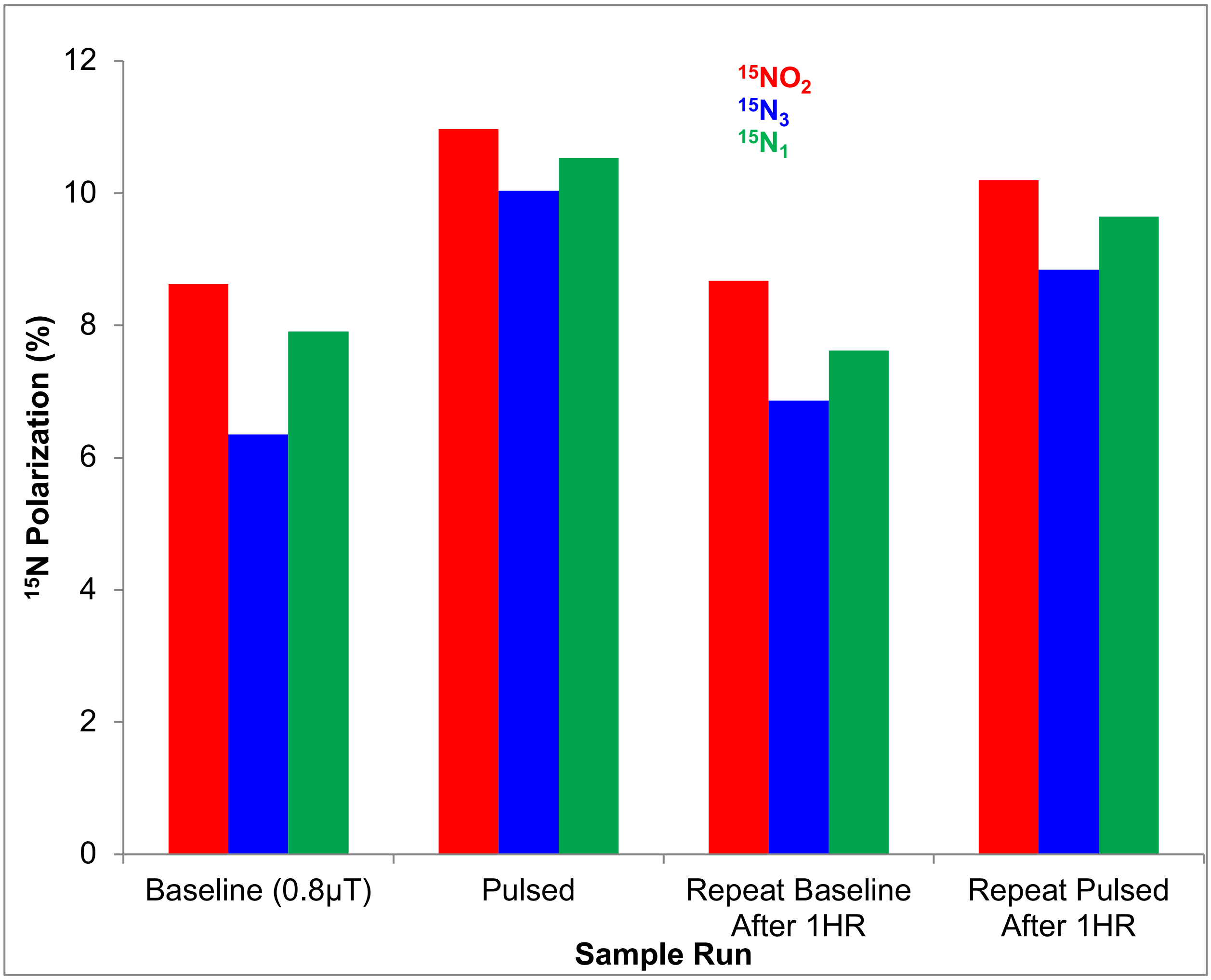

Figure S3. Comparison of the performance of pulsed SABRE-SHEATH method to static SABRE-SHEATH of [$^{15}N_3$]metronidazole using the sequence parameters shown in **Figure 3c** (B_{HIGH} = -28 μT, τ_{HIGH} = 1.7 ms, τ_{LOW} = 4 ms, B_{LOW} = 0.0 μT). Note that P_{15N} values are presented in the magnitude mode.

Table S1. Description of the selected data points in **Figure S3.** For the static field experiments, the field was that 0.8 μT, and for the pulsed field experiment, the sequence parameters were B_{HIGH} = -28 μT, τ_{LOW} = 4 ms, and B_{LOW} = 0.0 μT. Using the two series of repeat runs (baseline (static field) versus pulsed experiments, comparison of rows 1-4, P_{15N} improvement of pulsed versus static approach was quantified at (1.32±0.14)-fold improvement. Please, note that the magnitude of P_{15N} values are provided in the table.

**Sample Run**	**P_{15N} (%) $^{15}NO_2$**	**P_{15N} (%) $^{15}N_3$**	**P_{15N} (%) $^{15}N_1$**	**Data Point Description**
Static field of 0.8 μT (baseline)	8.6%	6.3%	7.9%	This data was acquired before τ_{high} pulse sweep experiment in **Figure 3c**
Pulsed field #1	11.0%	10.0%	10.5%	τ_{HIGH} = 1.7 ms, data point from **Figure 3c**
Repeat of the static field of 0.8 μT (repeat of the baseline experiment above approximately 1 hour after)	8.7%	6.9%	7.6%	This data was acquired after τ_{high} pulse sweep experiment in **Figure 3c**
Pulsed field #2 (repeat of the pulsed experiment #1 above approximately 1 hour after)	10.2%	8.9%	9.6%	τ_{HIGH} = 1.7 ms
Pulsed field #3	9.8%	8.3%	8.7%	τ_{HIGH} = 2.2 ms, data point from **Figure 3c**
Pulsed field #4 (repeat of the pulsed experiment #2 above approximately 0.6 hours after)	9.1%	8.1%	8.6%	τ_{high} = 2.2 ms

4. Example of P_{15N} calculations

Examples of ^{15}N signal enhancement and polarization calculations for the spectrum shown in **Figure 1b** are provided below:

$$\varepsilon_{15N} = \frac{37750.5063}{93.3184} \times \frac{12.4}{0.020} \times 1.52 = 3.80 \times 10^5$$

$$P_{15N} = 3.80 \times 10^5 \times 4.86 \times 10^{-5}\% = 18.5\%$$
